# Immunological diagnosis of CMV infection in HIV-infected patients

**DOI:** 10.1186/1758-2652-13-S4-P192

**Published:** 2010-11-08

**Authors:** IM Dumitru, S Rugina, E Dumitru, RC Cernat, S Diaconu, C Maxim Mitroi

**Affiliations:** 1 Ovidius University, Constanta, Romania

## Background

The Quantiferon-CMV assay is a test for Cell Mediated Immune (CMI) responses to peptide antigens that simulate CMV proteins. Individuals infected with CMV usually have CD8+ lymphocytes in their blood that recognize these antigens. This recognition process involves the generation and secretion of the cytokine and interferon (IFN). The detection and subsequent quantification of IFN forms the basis of this test.

## Purpose of the study

To evaluate the accuracy of Quantiferon-CMV assay in diagnosis of CMV infection in HIV positive patients and compare it with older ELISA method.

## Methods

This is a prospective study on a group of 48 HIV infected patients that are in a constant supervision of the Regional HIV Centre Constanta, Romania. The patients were tested by Quantiferon-CMV assay and by CMV ELISA. In the study were included adults patients with known HIV infection and CD4 < 200 cells/mm^3^, asymptomatic or with suggestive clinical manifestations: neurological (central or peripheral), ocular, gastrointestinal, pancreatic or hepatic symptoms. The patients with other associated opportunistic infections were excluded from the study.

## Results

Sixteen women and 32 men were included in the study, with a mean age of 32.8 years (range between 20-62 years), of whom three were antiretroviral naive patients and 45 were multiple experienced. Of these, seven patients were asymptomatic, 41 had different clinical manifestations: pancreatitis (12), esophagitis (3), gastritis (2) colitis (6), liver involvement (9), cholecystitis (7), central and peripheral nervous system involvement (8) retinitis (4). IgM ELISA was negative in all cases, IgG ELISA was positive in 45 patients (95.5%) and Quantiferon-CMV assay was reactive in 38 cases (79.1%). All patients with CD4 < 50 cells/mm^3^ with clinical manifestations showed both positive reactions (14 patients). The patients who were treated with gancyclovir for CMV retinitis showed positive ELISA IgG, but negative Quantiferon-CMV assay. The sensitivity of Quantiferon-CMV assay was 92.7%.

## Conclusions

Quantiferon-CMV assay has increased sensitivity in the detection of CMV reactivation induced by severe immunodepression (92.7%). There is a strong correlation (p < 0.01) between severe immunodepression (CD4 < 50 cells/mm^3^) and CMV reactivation detected by Quantiferon. This test is easy accessible and an efficient method of monitoring therapy with gancyclovir.

